# Cholestatic hepatitis as a possible new side-effect of oxycodone: a case report

**DOI:** 10.1186/1752-1947-2-140

**Published:** 2008-05-01

**Authors:** Vincent Ho, Maxwell Stewart, Peter Boyd

**Affiliations:** 1 School of Medicine, James Cook University, Cairns Base Hospital, Cairns, Queensland, 4870, Australia; 2 Department of Pathology, Cairns Base Hospital, Cairns, Queensland, 4870, Australia; 3 Department of Gastroenterology, Cairns Base Hospital, Cairns, Queensland, 4870, Australia

## Abstract

**Introduction:**

Oxycodone is a widely-used semisynthetic opioid analgesic that has been used for over eighty years. Oxycodone is known to cause side effects such as nausea, pruritus, dizziness, constipation and somnolence. As far as we are aware cholestatic hepatitis as a result of oxycodone use has not been reported so far in the world literature.

**Case presentation:**

A 34-year-old male presented with cholestatic jaundice and severe pruritus after receiving oxycodone for analgesia post-T11 vertebrectomy. Extensive laboratory investigations and imaging studies did not reveal any other obvious cause for his jaundice and a liver biopsy confirmed canalicular cholestatis suggestive of drug-induced hepatotoxicity. The patient's symptoms and transaminases normalised on withdrawal of oxycodone confirming that oxycodone was the probable cause of the patient's hepatotoxicity.

**Conclusion:**

We conclude that cholestatic hepatitis is possibly a rare side effect of oxycodone use. Physicians should be aware of the possibility of this potentially serious picture of drug-induced hepatotoxicity.

## Introduction

Medications are a common cause of hepatic injury which is not surprising as the liver is the predominant site of drug clearance, biotransformation and excretion. Oxycodone is a widely used semi-synthetic opioid that has been an effective analgesic agent for the last eighty years. Oxycodone has well-described side effects which include nausea, pruritus, dizziness, constipation and somnolence. Hepatic injury as a result of oxycodone use however has to date not been reported in the world literature.

We report a patient who experienced jaundice and pruritus after taking oxycodone after a T-11 vertebrectomy, with a clinical presentation and liver histology highly suggestive of oxycodone-induced cholestatic hepatitis.

## Case presentation

A 34-year-old man presented in May 2006 for an elective T11 vertebrectomy after a prior motor vehicle accident injury in 2004. His only pre-existing medication was Tramadol 200 mgs daily for analgesia. Pre-admission blood tests were normal.

At the time of operation he received 20 ml 0.5% Bupivacaine with adrenaline for local anaesthesia with 1 g Cephalothin administered for antibiotic prophylaxis. Propofol 860 mgs and Fentanyl 3000 micrograms were given as an infusion during and after the operation. Ketamine 600 mgs was administered via intravenous infusions for analgesia. The infusions were discontinued after 3 days. Blood tests at that point including liver enzymes were normal.

After the discontinuation of his pain infusions he was started on short acting oxycodone for analgesia. Three days after receiving a total of 40 mg of oxycodone, routine liver enzyme tests revealed bilirubin 8 μmol/L (<20 μmol/L), ALP 229 u/L (40–110 u/L), GGT 283 u/L (<50 u/L), ALT 78 u/L (<45 u/L) and AST 86 u/L (<40 u/L). He was asymptomatic. His liver dysfunction was attributed to the earlier combination of anaesthetic agents with analgesics and thought to be transient. Controlled release oxycodone was commenced and titrated with short acting oxycodone breakthrough to 40 mg oxycodone in the morning and 20 mg at night. He was discharged pain-free.

He represented to our hospital eight weeks later because of painless jaundice and debilitating generalised pruritus which had been present for the last two weeks. He had been taking only controlled release oxycodone at the doses of 40 mg in the morning and 20 mg at night every day since his prior hospital discharge. He had not taken any other medications since discharge including over-the-counter medications, herbal or traditional medicines. His admission bilirubin level was recorded at 140 μmol/L (<20 μmol/L), ALP 358 u/L (40–110 u/L), GGT 54 u/L (<50 u/L), ALT 295 u/L (<45 u/L) and AST 149 u/L (<40 u/L).

He had never consumed alcohol and there was no recent travel history. Viral serology for hepatitis and HIV were negative. An infectious screen was carried out as Far North Queensland has a high incidence of exotic illnesses that can cause transient liver enzyme derangement. CMV, EBV, Spotted fever, Scrub Typhus, Flavivirus, Dengue, Ross River, Barmah Forest and Leptospirosis serology were all unremarkable.

A liver screen carried out including serum copper and caeruloplasmin, α-fetoprotein, α-1 antitrypsin, iron studies, ANA, ANCA, anti-LKM1, anti-mitochondrial and anti-smooth muscle antibodies were unremarkable. Complete blood count was normal.

Abdominal ultrasound revealed the presence of a gallbladder full of calculi. No dilatation of the common bile duct or intrahepatic ducts was seen.

On CT cholangiogram the contrast material was noted to be excreted by the renal tract suggesting that the pathology lay at the hepatocellular level rather than ductal obstruction. A magnetic resonance cholangiopancreatography confirmed the presence of gallstones but was otherwise unremarkable with no ductal dilatation.

**Figure 1:**
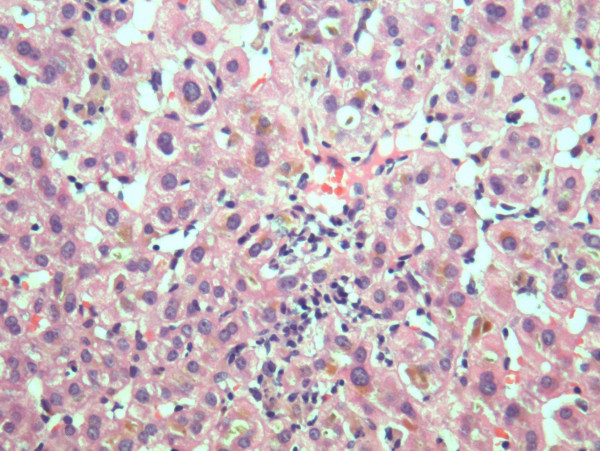
Liver biopsy image: bile thrombi (brown pigment) in dilated canaliculi is seen resulting in canalicular stasis.

A liver biopsy was performed and was striking for the presence of canalicular cholestasis with bile plugs in dilated canaliculi (figure 1). Occasional portal tracts contained a prominent lymphocytic infiltrate with mild piecemeal necrosis.

On withdrawal of oxycodone his liver function tests improved. One day after stopping his oxycodone his bilirubin level was 138 μmol/L (<20 μmol/L), ALP 188 u/L (40–110 u/L), GGT 34 u/L (<50 u/L), ALT 59 u/L (<45 u/L) and AST 53 u/L (<40 u/L).

One month after cessation his bilirubin level had fallen to 42 μmol/L (<20 μmol/L), ALP 140 u/L (40–110 u/L), GGT 34 u/L (<50 u/L), ALT 127 u/L (<45 u/L) and AST 100 u/L (<40 u/L). His liver function tests 6 months post-cessation of oxycodone are in the normal range and jaundice and pruritus have completely resolved.

## Discussion

Oxycodone is a widely used semi-synthetic opioid analgesic derived from the opium alkaloid thebaine. Compared with morphine, oxycodone has a higher oral bioavailability and is about twice as potent [1]. Oxycodone is metabolized by demethylation to noroxycodone and oxymorphone followed by glucuronidation [2].

Frequent side-effects of oxycodone include nausea, pruritus, dizziness, constipation and somnolence. Less frequent but serious side effects include hypotension and respiratory depression.

Cholestasis is associated with altered opioidergic neurotransmission and this is demonstrated through a number of lines of evidence. Firstly there is an opiate withdrawal-like reaction that patients with cholestasis can experience after the administration of opiate antagonists [3]. Secondly, increased plasma concentrations of some opioid peptides have been demonstrated in patients with cholestasis and in an animal model of cholestasis [4]. Hepatocytes have been shown to increase mRNA for met- and leu-enkephalins, suggesting the liver as a source of these opioids [5]. Finally the down-regulation of central opioid receptors has been shown in the brain of rats with cholestasis [6].

Morphine has been clearly linked to cholestatic pruritus and altered central opioidergic tone via the mu receptor pathway is thought to be a contributing cause [7]. That the pruritus of cholestasis is mediated, at least in part, by endogenous opioids, is supported by the observation that pruritus can be ameliorated by opiate antagonists. The precise mechanism for how morphine can cause cholestatic pruritus is however yet to be elucidated.

Oxycodone shares similar pharmacodynamic properties to morphine and displays binding to the mu-1 and kappa receptors, hence its use may result in cholestatic pruritus via increased central opioidergic tone [8].

To our knowledge no case of cholestatic hepatitis during oxycodone use has been reported. In our case the patient did have exposure to a number of anaesthetic and analgesic agents in his first admission which could have contributed to hepatotoxicity. However the persistence and exacerbation of his elevated liver enzymes and the manifestation of symptoms on oxycodone alone suggests that it is the probable cause of hepatotoxicity. Extrahepatic biliary pathology was deemed unlikely to result in his clinical picture in the absence of ductal dilatation on multiple imaging modalities. Liver biopsy showed features consistent with drug-induced inflammatory intrahepatic cholestasis and other diseases of the liver were excluded. Importantly, discontinuation of oxycodone led to a resolution of symptoms and a gradual but progressive return to normality on liver enzyme tests.

A Naranjo probability scale [9] utilised in our case scored 6 consistent with a probable adverse drug reaction. A more specific clinical scale for hepatotoxicity was likewise consistent with probable hepatotoxicity from oxycodone, with a RUCAM score of 7 [10]. Notification has been submitted to the Australian Adverse Drug Reactions Advisory Committee (ADRAC).

We did not consider rechallenging our patient with oxycodone due to ethical concerns as he was symptom free and there was a considerable risk of inducing fulminant hepatic failure.

## Conclusion

In summary, we report a potential case of cholestatic hepatitis as a consequence of oxycodone use. As far as we are aware this is the first report in literature to document possible hepatotoxicity from oxycodone use. As oxycodone is a widely used opioid analgesic physicians should be aware of the possibility of this rare but potentially serious adverse drug reaction.

## Competing interests

The authors declare that they have no competing interests.

## Authors' contributions

VH collated the information from the medical file and was the treating registrar. MS carried out the histopathological examination of the biopsy specimen. PB as the consultant-in-charge of the case made the provisional diagnosis of oxycodone-induced cholestatic hepatitis. All authors read and approved the final manuscript.

## Consent

Written informed consent was obtained from the patient for publication of study and any accompanying images. A copy of the consent is available for review by the Editor-in-Chief of this journal.
